# Novel Homozygous TTI2 Variant Causing Autosomal Recessive Syndromic Intellectual Disability and Primary Microcephaly from Pakistan: A Case Report (Exome Report)

**DOI:** 10.1155/2022/2766957

**Published:** 2022-08-12

**Authors:** Zul Qarnain, Fatima Khan, Fizza Akbar, Salman Kirmani

**Affiliations:** ^1^Medical College, The Aga Khan University, Karachi, Pakistan; ^2^Division of Women and Child Health, The Aga Khan University, Karachi, Pakistan

## Abstract

We describe a male patient with a novel TTI2 variant, which has not been previously associated with a human phenotype. His features include intellectual disability, primary microcephaly, delayed psychomotor development, speech delay, short stature, dysmorphic facial features, esotropia, kyphoscoliosis, and behavior abnormalities (Figure). Next generation sequencing revealed autosomal recessive TTI2 variant with uncertain significance, denoted as c.21_22insAAGCGCTCTG (p.Glu8Lysfs × 12). TTI2 encodes a regulator of DNA damage response and helps maintain steady levels of the PIKK family of protein kinases. No disease-causing variants in other genes potentially linked to his clinical presentation were identified. We report a novel loss-of-function homozygous variant in TTI2 that leads to syndromic intellectual disability and primary microcephaly.

## 1. Introduction

**Figure 1:**
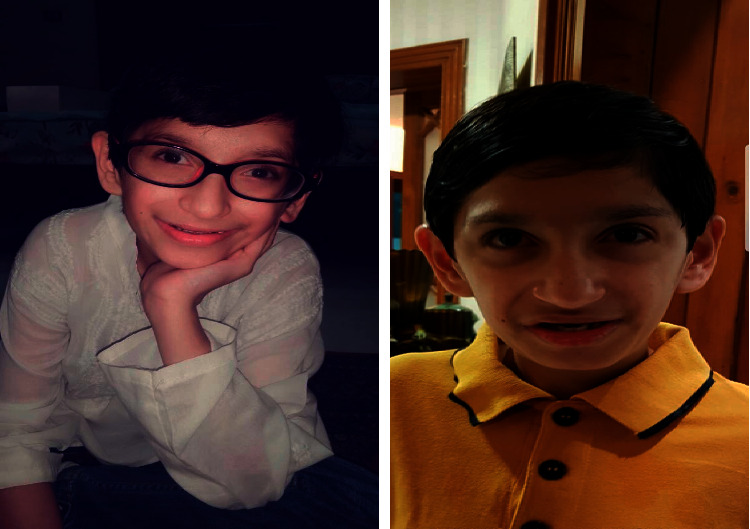
Dysmorphic features in the patient, including low set ears, prominent nose, deep-set eyes, and a relatively large mouth, consistent with those reported (MIM#615541) (https://www.omim.org/clinicalSynopsis/615541).

We describe a patient with dysmorphic facial features, syndromic intellectual disability, microcephaly, delayed speech and psychomotor development, short stature, esotropia, and kyphoscoliosis. At age 14 years, trio whole exome sequencing (WES) of this patient and his unaffected parents revealed a homozygous TTI2 variant, denoted as c.21_22insAAGCGCTCTG (p.Glu8Lysfs × 12) introducing a frame shift leading to a premature termination codon that results in a truncated protein product (Figure 1).

TTI2 (MIM#614426) encodes a regulator of DNA damage response that resides on *p* arm of chromosome 8 (8p12). Together with TELO2 and TTI1, these genes encode proteins that constitute the triple T-heterotrimeric complex (TTT complex), expressed strongly in developing brain together with TELO2 and TTI1, implicated in numerous cell functions. This complex is responsible for cellular resistance to DNA damage caused by ionizing and ultraviolet radiation and mitomycin C; it becomes a super-complex after interacting with HSP90 and helps maintain steady levels of phosphatidylinositol 3-kinase-related protein kinase (PIKK) family of proteins. PIKKs are involved in key cellular processes, including DNA breakage response, cellular proliferation, DNA replication stress, and growth response to nutrient availability [1–3]. Functional studies have shown that variants in both TTI2 and TELO2 result in a decrease in the stability of PIKKs [4–6].

The identified loss-of-function variant in our patient is likely to result in destabilizing the PIKK family of protein kinases leading to decreased DNA damage response; however, validation studies such as functional studies for this variant were not performed.

## 2. Clinical Report

**Figure 2:**
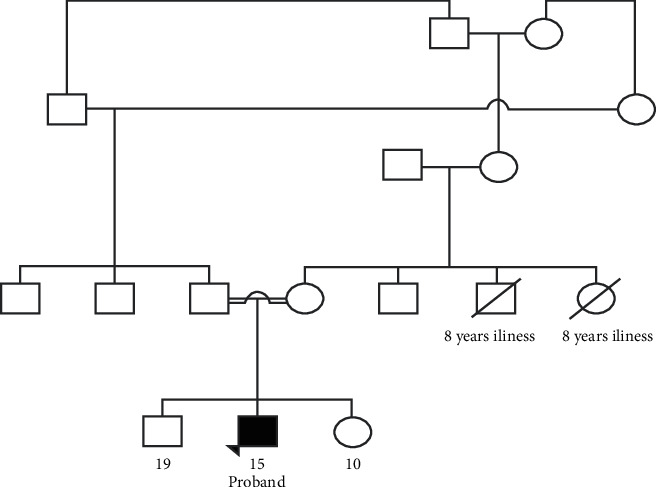
Pedigree of the patient showing consanguinity in the parents (drawn using online tool; progeny genetics) (https://pedigree.progenygenetics.com/).

The proband was born in August 2005 to a 22-year-old, gravida 1, para 1 mother via C-section at 36 weeks of gestation. The parents are consanguineous (Figure 2). There were no unusual exposures during pregnancy, and no complications were noted till the final trimester. Oligohydramnios and microcephaly were found on prenatal ultrasound at 35 weeks of gestation. Apgar scores at birth were 8 and 9, at 1 and 5 minutes, respectively, and there was no perinatal and postnatal distress. Neonatal examination was significant for microcephaly with head circumference of 29 cm (−2 S.D.) with a body weight of 2.2 kg and length of 48 cm.

Neck holding was achieved at six months. At eight months, his head circumference was 39 cm, weight was 8 kg, and height was 66 cm. At 17 months, his head circumference was 41 cm, weight was 8.1 kg, and height was 71 cm. At this point, his immunization profile was up to date and no feeding problem was reported, and the patient was able to sit at one year followed by bottom shuffling at 18 months and started walking at 3.5 years. During his developmental milestone assessment at four years of age, pincer grip was present, and he was unable to draw a circle or turn pages of books. He could not dress or undress himself without assistance and was not toilet trained. Moreover, at age of 15, he can say two-three word sentences with vocabulary of 40–50 words. His social development is appropriate for age. In addition, he is also enrolled in physiotherapy and occupational and speech therapy to improve his quality of life.

On examination in the Genetics Clinic at the age of 14 years, he had low set and prominent ears with microcephaly and open posterior fontanelle. He had deep-set eyes with a prominent nose and a relatively large mouth. He could write 1–20 digits and A–Z after continuous practice. He had difficulty in standing from a sitting or squatting position, but no visible wasting in any limb was noted. He had a flat foot (pes planus), and his toes point outwards while he walks.

Chest exam at age 14 was unremarkable with normal vesicular breathing bilaterally. Abdomen was soft and non-tender, and cardiovascular (CVS) exam was normal with S1 + S2 + 0 present. Central nervous system (CNS) exam showed developmental delay with speech being mainly affected. His past medical history identified scoliosis for which he underwent posterior instrumentation and correction of scoliosis deformity. Moreover, he has esotropia since birth, 6/16 and 6/36 in the right and left eyes, respectively. There is no history of congenital abnormalities in the close and extended family.

MRI of the proband was performed at 8 months of age. It showed normal brain parenchyma with no midline shift or any other structural abnormalities. EEG report at the age of 2 years and 5 months was also not significant. Extensive metabolic studies including serum ammonia, thyroid stimulating hormone levels, biotinidase, lactate, plasma amino acids, carnitine panel, complete electrolyte panel, organic acids, urine ketones, purine and pyrimidine panel, cerebrospinal fluid amino acids, and neurotransmitters were unrevealing.

At the age of 14, trio-based WES was sent to a clinical lab (Invitae Genetics, USA), which revealed a homozygous variant of uncertain significance in TTI2 (NM_001102401.2) c.21_22insAAGCGCTCTG (p.Glu8Lysfs × 12). The parents were both heterozygous carriers of this variant. No other pathogenic or likely pathogenic variants in other genes potentially linked to his phenotype were identified. The Human Phenotype Ontology (HPO) details used by the laboratory for analysis and prioritization of the genes included abnormal facial shape (HP: 0001999), global developmental delay (HP: 0001263), large face (HP: 0100729), moderate global developmental delay (HP: 00011343), moderately short stature (HP: 0008848), neurodevelopmental delay (HP: 0012758), scoliosis (HP: 0002650), short stature (HP: 0004322), and thoracolumbar kyphoscoliosis (HP: 0003423).

The released laboratory report has classified the variant as a variant of uncertain significance (VUS), based on the American College of Medical Genetics (ACMG) criteria. However, as we understand the role of TTI2 in connection with the neurodevelopmental phenotype spectrum, we believe that the identified variant is likely to be disease causing.

*Variant Details*. The sequence change creates a premature translational stop signal that is expected to result in a disrupted or absent protein product. The variant is not reported in population database (gnomAD) and applicable computational tools such as MutationTaster predict this insertion to be deleterious, and the reported phenotype in our patient is consistent with previous reports [7, 8]. A loss-of-function variant has been reported to be disease causing [9]. Therefore, we propose a reclassification of this variant as pathogenic according to ACMG criteria [10] (ACMG criteria scores PVS1, PM2, PM4, and PP4).

## 3. Methodology

### 3.1. Editorial Policies and Ethical Consideration

The Aga Khan University Hospital's ethics committee approved this case report for publication (2021-6062-20033). The case was discussed with patient's parents and written documented consent was taken from the parents for inclusion of unmasked pictures and videos of the proband. Patient's name and other family details will remain confidential.

### 3.2. Methods

Next generation sequencing (NGS), particularly trio-based whole exome sequencing, has led to the detection of de novo and inherited variants causing both syndromic and non-syndromic intellectual disability (ID).

DNA samples were obtained from proband and parents and were sent to Invitae Genetics Lab in the United States. Genomic DNA obtained from submitted sample was enriched for targeted regions using a hybridization-based protocol and sequenced using Illumina technology. All targeted regions are sequenced to an average of ≥50x depth with a minimum call depth of ≥20x. Reads are aligned to a reference sequence, and sequence changes are identified and interpreted in the context of a single clinically relevant transcript.

## 4. Discussion

Intellectual disability (ID) is a common neurodevelopmental disorder with a prevalence of 10.4/1000 and carries a major psychosocial and socioeconomic burden [11, 12]. Microcephaly is described as a reduction in fronto-occipital head circumference and is categorized as primary microcephaly when it is present before 36 weeks of gestation. Primary microcephaly can be caused by viral infections, exposure to alcohol and drugs, or due to an underlying genetic etiology. It is a rare disorder with the incidence of under 10/25000 live births and is associated with over 10 genes, but in majority of the cases, the real cause of primary microcephaly remains unclear [13–16]. Next generation sequencing (NGS) has led to great progress in deciphering monogenic causes of intellectual disability, particularly trio-based whole exome sequencing, which has led to the detection of de novo and inherited variants causing both syndromic and non-syndromic ID and microcephaly [17, 18].

We identified the first case from Pakistan with a novel homozygous TTI2 variant c.21_22insAAGCGCTCTG (p.Glu8Lysfs × 12) and features including intellectual disability, primary microcephaly, delayed psychomotor development, speech delay, short stature, dysmorphic facial features, esotropia, kyphoscoliosis, and behavior abnormalities.

A literature search found previous TTI2 variants that we discuss in chronology from most recent to earlier reported variants, summarized in Table 1. In 2020, a single French Canadian case born from second cousin heterozygous parents was reported with homozygous TTI2 variant c.950A > T (p.Asp317Val) presenting with microcephaly and short stature but no intellectual disability [19]. Earlier in 2019, two groups of compound heterozygous TTI2 variants were described with intellectual disability and microcephaly; however, none of the reported individuals were homozygous for this variant [9, 20]. Previously another TTI2 variant c.1307T > A(p.Ile436Asn) was described among three siblings born from consanguineous parents; contrary to our case, all the siblings had normal neonatal period and presented with progressive microcephaly at adult age and cognitive impairment [5]. An additional earlier larger Iranian consanguineous family study was also described in 2011 with c.1100C > T p.Pro367Leu (P367L) TTI2 gene variant presenting with non-syndromic intellectual disability [4].

Disease databases including Leiden Open Variation Database (LOVD) and ClinVar report a total of 47 variants. LOVD has reported 10 variants in TTI2, eight of them being missense, one being synonymous, and one being intronic variant; two are classified as pathogenic, three as likely pathogenic, two as a VUS, two as likely benign, and one as benign [21]. ClinVar database records a total of 37 variants in TTI2; out of them only one variant is classified as pathogenic, 16 are VUSs (including the variant reported in our patient by Invitae), and 20 variants are classified as likely benign while five are classified as benign [22]. About half of these reported variants have been classified as VUS in the databases, necessitating further work in delineating TTI2-associated phenotype spectrum complemented by functional studies to better understand the variant contribution in disease manifestation.

## 5. Conclusion

In conclusion, we described the first case from Pakistan with a novel homozygous TTI2 pathogenic variant (c.21_22insAAGCGCTCTG; p.Glu8Lysfs × 12), presenting with intellectual disability and primary microcephaly. No pathogenic variants in other genes potentially linked to his phenotype were identified, through trio WES testing. This further highlights the importance of TTI2 in the TTT complex, where it plays a functional role in maintaining the PIKK family of protein kinases and hence helps in DNA damage response and neurogenesis. It further stresses that certain probable homozygous TTI2 variants might present with both syndromic intellectual disability and primary microcephaly, supporting the need to include TTI2 in targeted NGS panels of intellectual disability and primary microcephaly.

## Figures and Tables

**Table 1:** TTI2 reported variants.

TTI2 variant	Amino acid changes	Zygosity	Phenotype	Reported cases	Publication year	References
c.21_22insAAGCGCTCTG	p.Glu8Lysfs × 12	Homozygous	Intellectual disability, microcephaly, delayed psychomotor development, speech delay, short stature, dysmorphic facial features, esotropia, kyphoscoliosis, and behavior abnormalities	1	Reported here	Reported here

c.950A > T	p.Asp317Val	Homozygous	Primary microcephaly, short stature, severe speech delay, dysmorphic features, strabismus, and dyskinesia	1	2020	[19]

Patient 1 c.1075C > T and c.950A > T	p.Arg359Cys and p.Asp317Val	Compound heterozygous	Intellectual disabilities, progressive microcephaly, high nasal bridge, deep-set eyes, and partial ovarian failure	2	2019	[20]
Patient 2 c.539T > C and c.575T > C	p.Leu180Pro and p.Leu192Pro	Compound heterozygous				

c.942_944delTCTins and c.1100C > T	p.Leu315CysfsTer8 and p.Pro367Leu	Compound heterozygous	Intellectual disabilities, microcephaly, growth retardation, speech disorder, and movement disorders	2	2019	[9]

c.1307T > A	p.Ile436Asn	Homozygous	Normal growth parameters, microcephaly at adult age, severe cognitive impairment, severe speech delay, short stature, dysmorphic features, and vertebral anomalies	3	2013	[5]

c.1100C > T	p.Pro367Leu	Homozygous	Non-syndromic moderate intellectual disability	2	2011	[4]

## Data Availability

The data that support the findings of this study are available on request from the corresponding author. The data are not publicly available due to privacy or ethical restrictions.

## Supplementary Materials

Supplementary Materials The patient is socially receptive to name calling. He is able to follow simple commands; however, execution is rather delayed. He often smiles while interacting with people and is oriented to space and people, especially his family members and teachers. The patient can also identify objects. He is able to engage emotionally with family members, i.e., hugging. He has a characteristic gait with outward pointing feet. The patient's video in supplementary files can be accessed to validate the above commentary.
