# Supplementary material for: Novel Homozygous TTI2 Variant Causing Autosomal Recessive Syndromic Intellectual Disability and Primary Microcephaly from Pakistan: A Case Report (Exome Report)
Source: Case Rep Genet. 2022 Aug 12;2022:2766957. doi: 10.1155/2022/2766957 (PMC9391182; doi:10.1155/2022/2766957)
Supplement: Supplementary Materials — The patient is socially receptive to name calling. He is able to follow simple commands; however, execution is rather delayed. He often smiles while interacting with people and is oriented to space and people, especially his family members and teachers. The patient can also identify objects. He is able to engage emotionally with family members, i.e., hugging. He has a characteristic gait with outward pointing feet. The patient's video in supplementary files can be accessed to validate the above commentary. [file 2766957.f1.docx]

<https://drive.google.com/file/d/1IwN0xkhFjBIyZi01ejCeAi2h63piUh6y/view?usp=sharing>

<https://drive.google.com/file/d/19E9fOnNV37eyhA3FOjjigPP0R7Ita7jS/view?usp=sharing>

*The above links given show videos of the patient’s phenotype*
